# CXCL1-CXCR2 signalling mediates hypertensive retinopathy by inducing macrophage infiltration

**DOI:** 10.1016/j.redox.2022.102438

**Published:** 2022-08-13

**Authors:** Shuai Wang, Jie Bai, Yun-Long Zhang, Qiu-Yue Lin, Xiao Han, Wei-Kun Qu, Peng-Fei Zhang, Yu-Song Ge, Qi Zhao, Hui-Hua Li

**Affiliations:** a Department of Ophthalmology, Second Affiliated Hospital of Dalian Medical University, Dalian, 116023, China; b Department of Emergency Medicine, Beijing Chaoyang Hospital, Capital Medical University, Beijing, 100020, China; c Department of Nutrition and Food Hygiene, School of Public Health, Dalian Medical University, Dalian, 116004, China; d Institute of Cardiovascular Diseases, First Affiliated Hospital of Dalian Medical University, Dalian, 116000, China; e School of Optoelectronic Engineering and Instrumentation Science, Dalian University of Technology, Dalian, 116024, China

**Keywords:** Hypertensive retinopathy, CXCL1, CXCR2, Macrophages, Inflammation

## Abstract

Inflammation plays an important role in hypertensive retinal vascular injury and subsequent retinopathy. Monocyte chemotaxis via CXCL1-CXCR2 binding has been implicated in various cardiovascular diseases, but the function of CXCL1-CXCR2 signalling involved in retinopathy, which was investigated as angiotensin II (Ang II)-induced retinopathy, is unclear. In our study, we established a hypertensive retinopathy (HR) model by Ang II infusion (3000 ng/min/kg) for 3 weeks. To determine the involvement of CXCR2 signalling, we used CXCR2 knockout (KO) mice or C57BL/6J wild-type (WT) mice as experimental subjects. The mice were treated with a CXCL1 neutralizing antibody or SB225002 (the specific CXCR2 inhibitor). Our results showed that after Ang II treatment, the mRNA levels of CXCL1 and CXCR2 and the number of CXCR2^+^ inflammatory cells were significantly elevated. Conversely, unlike in the IgG control group, the CXCL1 neutralizing antibody greatly reduced the increase in central retinal thickness induced by Ang II infusion, arteriolar remodelling, superoxide production, and retinal dysfunction in WT mice. Furthermore, Ang II infusion induced arteriolar remodelling, infiltration of Iba1^+^ macrophages, the production of oxidative stress, and retinal dysfunction, but the symptoms were ameliorated in CXCR2 KO mice and SB225002-treated mice. These protective effects were related to the reduction in the number of CXCR2^+^ immune cells, particularly macrophages, and the decrease in proinflammatory cytokine (IL-1β, IL-6, TNF-ɑ, and MCP-1) expression in Ang II-treated retinas. Notably, serum CXCL1 levels and the number of CXCR2^+^ monocytes/neutrophils were higher in HR patients than in healthy controls. In conclusion, this study provides new evidence that the CXCL1-CXCR2 axis plays a vital role in the pathogenesis of hypertensive retinopathy, and selective blockade of CXCL1-CXCR2 activation may be a potential treatment for HR.

## 1 Introduction

Hypertension is a risk factor for various retinal diseases, including hypertensive retinopathy, glaucoma and macular degeneration [1]. HR typically involves sequential phases of vasoconstriction, exudation, and hardening, followed by complications of hardening. Generally, hypertension influences the eyes in multiple ways, including by inducing the development of retinopathy, choroidopathy, and optic neuropathy [1]. Despite the considerable attention given to hypertension and its complications, the pathogenesis of HR, especially the initiation of lesion formation, is not yet completely understood. Growing evidence suggests that HR occurrence and development can be attributed to the inflammatory response [2]. Various hypertensive stimuli, such as angiotensin II (Ang II) and reactive oxygen species (ROS), can induce the migration and infiltration of proinflammatory cells, including mononuclear/macrophages, neutrophils, and T cells, into the retina. Interestingly, mononuclear macrophages contribute to Ang II-induced hypertension and vascular dysfunction [3,4]. Our recent studies further demonstrated that increased macrophage recruitment and superoxide production in the retina are closely associated with Ang II-induced retinopathy [5,6]. Indeed, activated macrophages release a large number of proinflammatory cytokines (IL-1β, IL-6, and TNF-ɑ) and a large amount of ROS, which are vital for retinal inflammation and retinopathy [[5], [6], [7]]. However, the precise mechanism by which Ang II promotes macrophage infiltration into the retina to cause HR remains unclear.

In recent years, studies have demonstrated that chemokines and their receptors are critical for the regulation of immune cell migration and the inflammatory response. Chemokines are a large class of small molecules with chemoattractant properties that can drive the recruitment of leukocytes through binding with their cognate receptors, which are mainly divided into four groups, i.e., CCRs (CCR1-10), CXCRs (CXCR1-7), CXCR1 and CX3CR1. Chemokines, including CCL2, CCL4, CXCL9, and CXCL10, can be detected in the eyes of diabetic retinopathy (DR) patients [8]. Among the chemokines, CXC chemokines, including CXCL1, CXCL2, CXCL3, CXCL5, CXCL6, CXCL7 and CXCL8, recruit immune cells by binding with CXCR2. CXCL1-CXCR2 signalling is involved in myocardial I/R injury, hypertension, cardiac remodelling, and the ventricular fibrillation induced by Ang II [9,10]. Interestingly, a recent study reported that CXCL1 is crucial for retinal leukocyte recruitment and ischaemia/reperfusion (I/R)-induced retinopathy [11]. CXCL8 (also known as IL-8) levels were found to be highly upregulated and significantly increased the Ca^2+^-dependent outflow of K^+^ in red blood cells in patients with hypertension [12] Moreover, CXCL5-CXCR2 signalling mediates lens injury-induced cell infiltration through AKT activation [13]. However, the role of the CXCL1-CXCR2 axis in Ang II-induced monocyte recruitment to the retina and retinopathy is unclear.

To explore the function of the CXCL1-CXCR2 axis in Ang II-induced retinopathy, we used CXCR2 knockout (KO) mice and C57BL/6J wild-type (WT) mice as experimental subjects. The mice were given either a CXCL1-neutralizing antibody or a specific inhibitor of CXCR2 (SB225002). Our data indicated that with the pharmacological blockade of CXCL1 or CXCR2 or CXCR2 deletion, the increase in central retinal thickness, arterial narrowing, infiltration of Ib1a^+^ macrophages, ROS generation, and retinal dysfunction were significantly ameliorated in Ang II-treated mice. Such beneficial effects were related to the reduction in the number of CXCR2^+^ immune cells, particularly macrophages, and the decrease in proinflammatory cytokine (IL-1β, IL-6, TNF-ɑ, and MCP-1) expression in Ang II-treated retinas. This study provides new evidence that the CXCL1-CXCR2 axis plays a critical role in the pathogenesis of hypertensive retinopathy, and selective blockade of CXCL1-CXCR2 activation may be a potential treatment for HR.

## 2 Results

### 2.1 mRNA levels of CXCL1 and CXCR2 and the number of CXCR2^+^ myelomonocytes are elevated in retinas following Ang II infusion

**Fig. 1:**
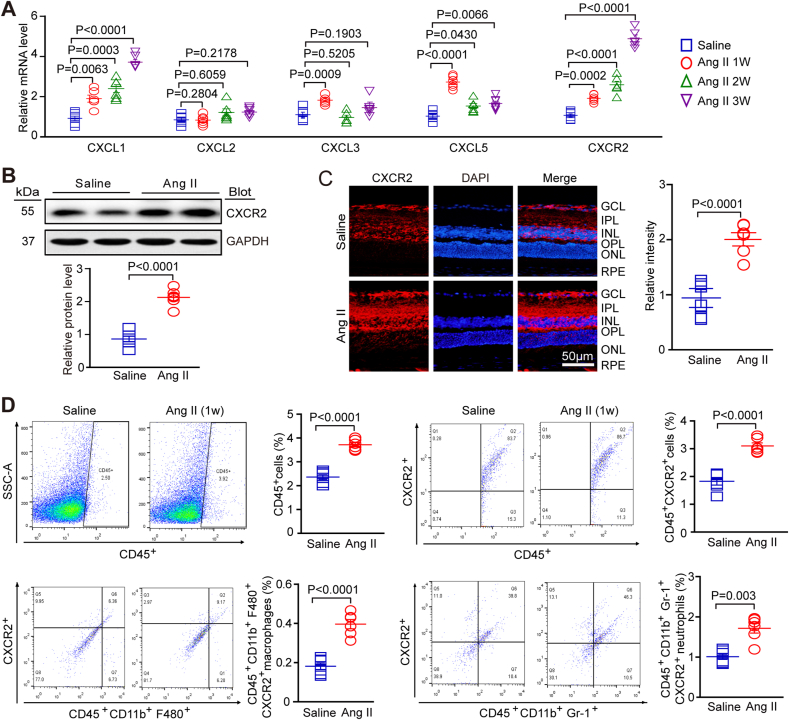
**Ang II infusion promoted the upregulation of CXCL1 and CXCR2 expression and increased the number of myeloid-derived CXCR2-positive cells in retinas.** (A) WT C57BL/6J mice were infused with Ang II or saline for 1, 2 and 3 weeks. Quantitative real-time polymerase chain reaction analyses of chemokine mRNA and CXCR2 mRNA expression in the retina (n = 6). (B) The expression of CXCR2 protein (right, upper) was analysed by Western blot, and the protein levels (right, lower; n = 6 in each group) were quantified. GAPDH was used as the internal control (n = 6). (C) Immunostaining of CXCR2 in retinal slices (left) with an anti-CXCR2 antibody and the quantification of the red fluorescence intensity in each group (middle; n = 6 in each group); (D) Flow cytometry analyses of CD45^+^ cells, CD45^+^ CXCR2^+^ cells, CD45^+^ CD11b^+^ F480^+^ CXCR2^+^ macrophages, and CD45^+^ CD11b^+^ Gr-1^+^ CXCR2^+^ neutrophils in the retina at one week after Ang II infusion. Percentage of each type of cell (right, n = 6). The data are presented as the mean ± SEM. (For interpretation of the references to colour in this figure legend, the reader is referred to the Web version of this article.)

We evaluated the expression levels of a series of chemokines (CXCL1, CXCL2, CXCL3 and CXCL5) and their receptor CXCR2 in Ang II-treated retinas to investigate the role of chemokines and their receptors in hypertensive retinopathy. At 1, 2 and 3 weeks after Ang II infusion, qPCR analyses showed that the mRNA levels of CXCL1 and CXCL5 in retinal tissue were higher in Ang II-infused mice than in saline-infused mice at different time points, as shown in Fig. 1A. The mRNA expression of their receptor, CXCR2, also increased over time in Ang II-treated retinas (Fig. 1A). Western blot analysis confirmed that the expression of CXCR2 was increased at the protein level (Fig. 1B). Immunohistochemical staining showed that CXCR2 was mainly expressed in the ganglia cell layer (GCL), inner plexiform layer (IPL) and inner nuclear layer (INL) of the retina; after Ang II treatment, there was also an increase in levels in the outer layers (Fig. 1C). To determine whether the increase in CXCR2 expression was associated with CXCR2^+^ immune cell recruitment to the retina, we performed flow cytometry and found that the number of CD45^+^ CXCR2^+^ cells, including CD11b^+^F480^+^CXCR2^+^ macrophages and CD11b^+^Gr-1^+^CXCR2^+^ neutrophils, was significantly higher in the Ang II-treated group than in the saline control group one week after infusion (Fig. 1D). Above all, these results suggest that Ang II infusion could upregulate the expression of CXC chemokines, leading to the increased infiltration of circulating CXCR2^+^ myeloid cells into the retina, which may be associated with retinopathy.

### 2.2 Application of a CXCL1-neutralizing antibody prevents Ang II-induced retinopathy

**Fig. 2:**
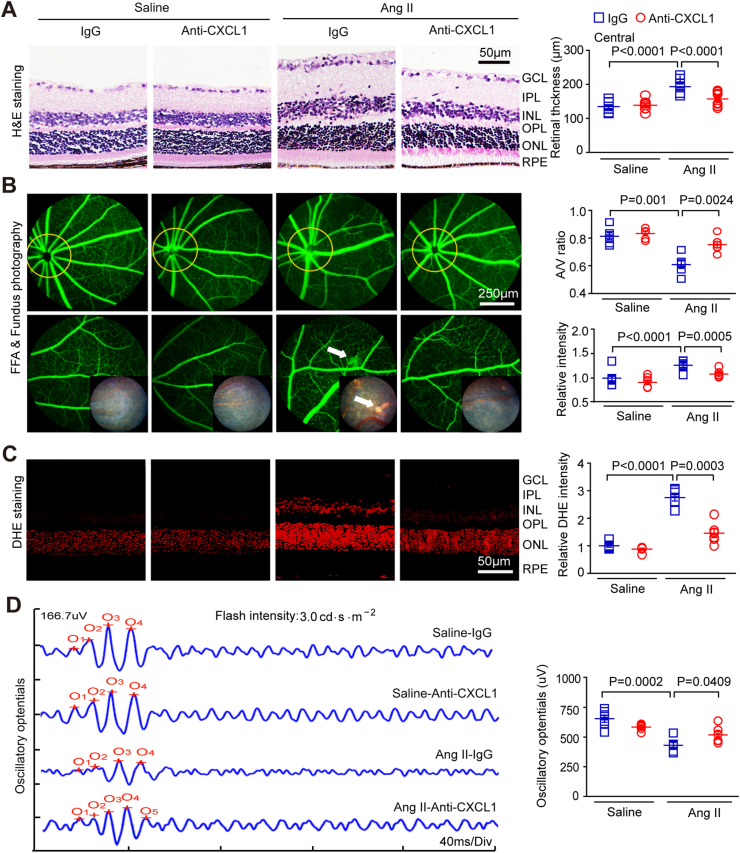
**Treatment with CXCL1 neutralizing antibody reduced retinal thickening, inflammation, ROS, and vascular dysfunction in Ang II-infused mice.** (A) WT C57BL/6J mice were treated with IgG control or CXCL1 neutralizing antibody (anti-CXCL1, 100 μg/mouse/day) and then infused with Ang II for three weeks. Images of H&E staining of central retinal sections (left) and quantitation of the retinal thickness (right, n = 8). (B) Typical retinal angiograms and fundus photos (left); these white arrows indicate vascular fluorescein leakage and the corresponding area in the fundus photo. The ratio of retinal arteriovenous and fluorescence intensity was quantified (right; n = 6). (C) Retinal superoxide production was evaluated by DHE staining (left); the red fluorescence intensity of DHE was quantified (right; n = 6). (D) Waveforms of oscillatory potential (left) and amplitude quantification (right; n = 6). (For interpretation of the references to colour in this figure legend, the reader is referred to the Web version of this article.)

Given that CXCL1 showed the most significant increase in expression, mainly following Ang II infusion (Fig. 1A), we first detected CXCL1 levels in the retinas of WT mice after daily administration of a CXCL1-neutralizing antibody (anti-CXCL1, 100 μg/mouse/day). The ELISA results showed that systemic injection of mice with anti-CXCL1 led to significantly lower CXCL1 levels in the retina of mice than injection with IgG after saline or Ang II infusion (Fig. S1A). Systolic blood pressure (SBP) was significantly increased in both control IgG-treated WT mice and anti-CXCL1-treated mice in response to Ang II (3000 ng/kg/min) stimulation for 3 weeks, but there was no statistically significant difference between the two groups (Fig. S1B). Furthermore, Ang II significantly increased the inner layer thickness of the central region of the retina, including in the GCL, IPL and INL, while the inner layer thickness of the retina was significantly decreased in mice in the anti-CXCL1 treatment group (Fig. 2A). Moreover, Ang II-induced impairment of the retinal arteriolar structure, as indicated by arteriolar narrowing (decreased artery-to-vein (A/V) ratio), tortuosity and exudation, was markedly better in the retinas of anti-CXCL1-treated mice than in those of IgG-treated controls (Fig. 2B).

Since oxidative stress is a hallmark of retinopathy, we next examined the effect of anti-CXCL1 treatment on ROS production with DHE (dihydroethidium) staining. Ang II led to a significant increase in superoxide generation and the mRNA expression of NADPH oxidases (NOX1 and NOX4) in the retinas of IgG-treated mice, which was remarkably attenuated in anti-CXCL1-treated mice (Fig. 2C, Fig. S1C). Oscillatory potentials (OPs) are an early indicator of mild retinal circulation disorder in HR [14]. We therefore performed electroretinography (ERG) to assess the functional status of the retina. Infusion of Ang II led to significantly lower OP amplitude in the retinas of mice treated with IgG than saline infusion [6]. Conversely, this decrease in OP amplitude was markedly rescued in anti-CXCL1-treated mice unlike in IgG-treated mice (Fig. 2D). Since the a-wave represents the activity of photoreceptors, the b-wave reflects the activity of on-bipolar cells. We then quantified the amplitude and timing of these waves in the retinas and found that Ang II infusion caused a significant decrease in b-wave amplitude in IgG-treated mice, but this effect was not markedly reversed in anti-CXCL1-treated mice (Fig. S1D). Additionally, there was no significant difference in the timing of the b-wave or the amplitude and timing of the a-wave between IgG- and anti-CXCL1-treated mice after saline of Azng II infusion (Figs. S1E–G). After saline infusion, no significant difference was observed regarding retinal thickness, ROS production, or vascular dysfunction between the two groups (Fig. 2A–D). Overall, the above findings suggest that CXCL1 contributes to Ang II-evoked retinopathy and dysfunction.

### 2.3 CXCR2 deficiency ameliorates retinopathy and dysfunction in Ang II-infused mice

**Fig. 3:**
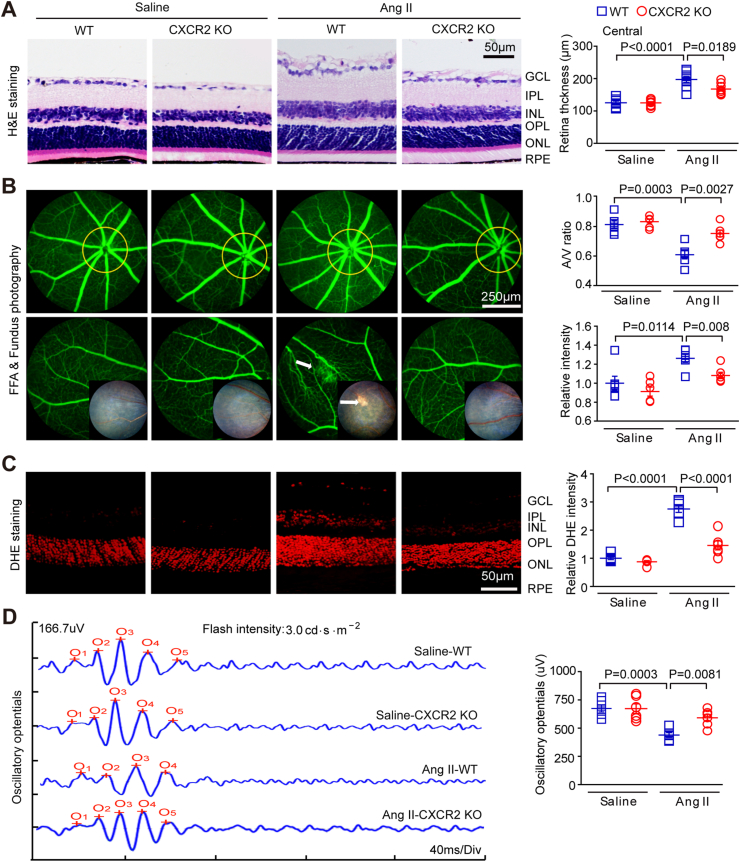
**Genetic ablation of CXCR2 ameliorates Ang II-induced retinopathy in mice.** (A) WT C57BL/6J mice and CXCR2 KO mice were both infused with angiotensin II or saline for three weeks. H&E-stained central retina sections (left). Retinal thickness was quantified (right, n = 8–10). (B) Representative angiograms and fundus photographs of retinal vessels (left); the arrows indicate leakage of fluorescein perivascularly and the corresponding fundus photograph area. The arteriovenous ratio and quantification of fluorescence intensity (right, n = 6). (C) DHE red fluorescence staining for analysis of superoxide production in the retinas of each group (left) and intensity quantification (right, n = 6). (D) Typical waveforms of oscillatory potentials and their amplitude quantification (n = 6). (For interpretation of the references to colour in this figure legend, the reader is referred to the Web version of this article.)

To determine the functional role of CXCR2 in HR development, WT and CXCR2 KO mice were infused with Ang II (3000 ng/kg/min) for 21 days. After Ang II infusion, the SBP in the WT group and CXCR2 KO mice was significantly higher than that in the saline group but was similar between the WT and CXCR2 KO groups (Fig. S2A). Interestingly, Ang II-induced central retinal thickening, arterial stenosis (A/V), distortion and exudation were significantly alleviated in CXCR2 KO mice (Fig. 3A–B). To test whether Ang II-induced thickening of the retina was associated with increased vascular leakage and retina oedema, we further detected the expression of vascular leakage marker VEGF-A and tight junction protein zonula occludens-1 (ZO-1) with immunostaining. Our results indicated that Ang II infusion resulted in markedly higher VEGF-A expression and lower ZO-1 expression in WT retinas than saline infusion, whereas these effects were significantly reversed in CXCR2 KO retinas (Figs. S2B–C). In addition, Ang II increased superoxide production in WT mice, and both NOX1 and NOX4 mRNA levels were significantly decreased in CXCR2 KO mice (Fig. 3C, Fig. S2D). Therefore, the decrease in the retinal OP amplitude in WT mice infused with Ang II was also alleviated (Fig. 3D). After infusion of normal saline, these retinal parameters were similar between the two groups (Fig. 3A–D). Thus, CXCR2 ablation can ameliorate Ang II-induced retinopathy.

### 2.4 Inhibition of CXCL1 or CXCR2 reduces Ang II-induced CXCR2^+^ inflammatory cell infiltration in retinas

**Fig. 4:**
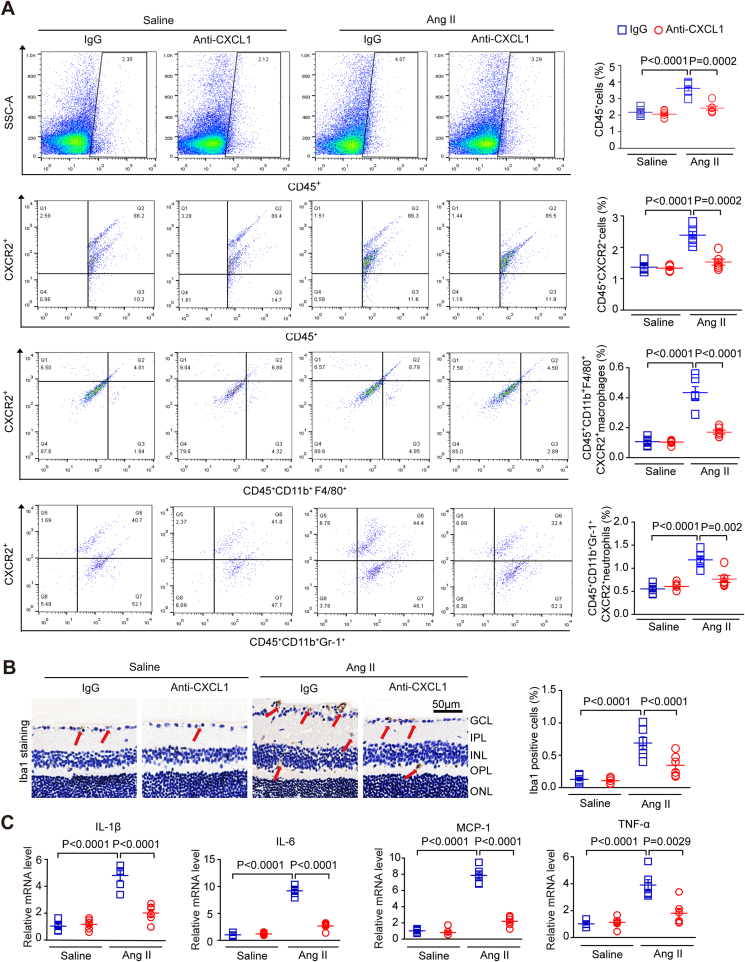
**Treatment with a CXCL1-neutralizing antibody prevents Ang II-induced inflammatory cell infiltration.** (A) WT C57BL/6J mice were pretreated with IgG control or anti-CXCL1 neutralizing antibody (100 μg/mouse/day) and then infused with angiotensin II for three weeks. Flow cytometry analyses of CD45^+^ cells, CD45^+^ CXCR2^+^ cells, CD45^+^ CD11b^+^ F480^+^ CXCR2^+^ macrophages and CD45^+^ CD11b^+^ Gr-1^+^ CXCR2^+^ neutrophils cells in each group (left). The percentage of gated cells among total cells (right, n = 6); (B) Representative images of Iba1 IHC staining in each group, red arrows indicate the Iba1-positive microglia/macrophages(scale bar: 50 μm, left); quantification of positive cells (right; n = 6 per group); (C) qPCR analyses of the mRNA levels of IL-1β, IL-6, tumour necrosis factor-α (TNF-α), and monocyte chemoattractant protein-1 (MCP-1) in each group (n = 6). GAPDH was used as an internal control. (For interpretation of the references to colour in this figure legend, the reader is referred to the Web version of this article.)

To elucidate the potential mechanism underlying the involvement of the CXCL1-CXCR2 axis in retinal hypertensive retinopathy, we first examined the effects of CXCL1 resistance on CXCR2^+^ cell infiltration into the retina. Flow cytometry results showed that the number of CD45^+^ CXCR2^+^ myeloid cells (CD11b^+^F480^+^CXCR2^+^ macrophages and CD11b^+^Gr-1^+^CXCR2^+^ neutrophils) was markedly upregulated in the retinas of control IgG-treated mice after Ang II infusion, but this effect was dramatically reduced in the retinas of anti-CXCL1-treated mice (Fig. 4A). Immunohistochemistry showed that Ang II increased the infiltration of Iba1^+^ macrophages. In contrast, the effect was markedly rescued in the retinas of CXCL1-treated mice, as shown in Fig. 4A–B. Accordingly, after Ang II infusion, the mRNA levels of cytokines, including interleukin-1β (IL-1β), IL-6, tumour necrosis factor (TNF-α), and monocyte chemoattractant protein-1 (MCP-1), were significantly higher than those in anti-CXCL1-treated mice (Fig. 4C).

**Fig. 5:**
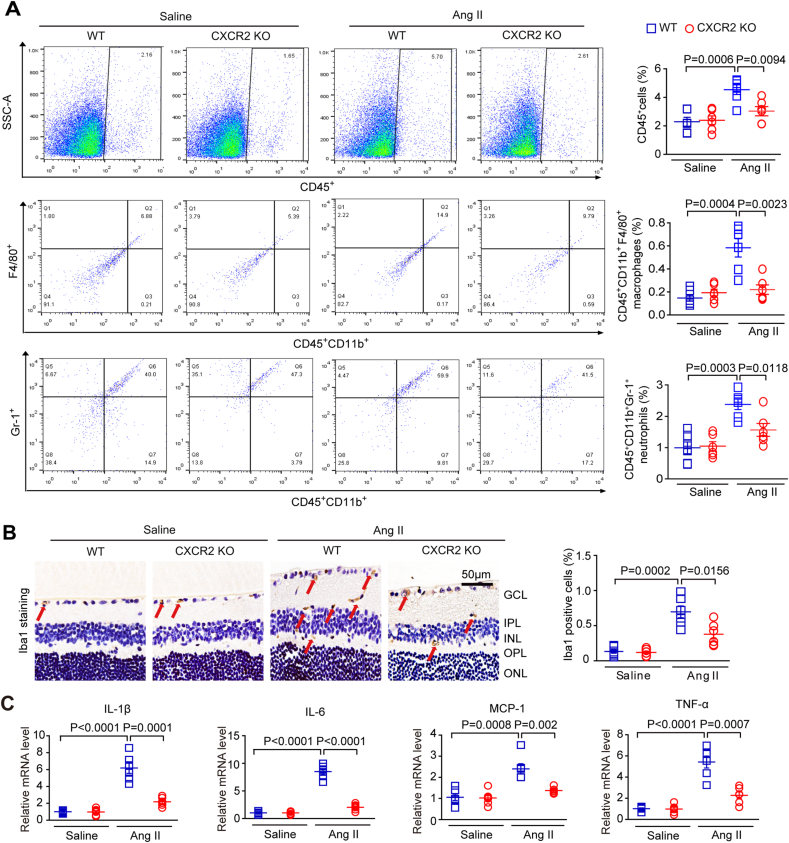
**Knockout of CXCR2 attenuates inflammatory cell infiltration induced by Ang II.** (A) Wild-type (WT) and CXCR2 KO mice were infused with Ang II (3000 ng/min/kg) or saline for 3 weeks. Flow cytometry analyses of CD45^+^ cells, CD45^+^ CD11b^+^ F480^+^ macrophages and CD45^+^ CD11b^+^ Gr-1^+^ neutrophils cells in retinas (left). The percentage of gated cells among total cells (right, n = 6). (B) Representative images of Iba1 IHC staining, red arrows indicate the Iba1-positive microglia/macrophages(scale bar: 50 μm, left); quantification of positive cells in each group (right, n = 6). (C) qPCR analyses of the mRNA levels of IL-1β, IL-6, TNF-α, and MCP-1 in the retinas (n = 6). GAPDH was used as an internal control. (For interpretation of the references to colour in this figure legend, the reader is referred to the Web version of this article.)

To further verify the effect of CXCR2 KO on the infiltration of proinflammatory cells in the retinas of mice, we performed flow cytometry, immunohistochemical staining, and qPCR analysis in WT and CXCR2 KO mice infused with Ang II (3000 ng/min/kg) or saline for 3 weeks. Consistent with the findings in anti-CXCL1-treated mice (Fig. 5A–C), Ang II-induced increases in the numbers of CD45^+^CD11b^+^F480^+^ macrophages, CD45^+^CD11b^+^Gr-1^+^ neutrophils, and Iba1^+^ macrophages and the mRNA levels of IL-1β, IL-6, TNF-α and MCP-1 in the retinas of WT mice were all significantly suppressed in the retinas of CXCR2 KO mice (Fig. 5A–C). There was no difference in these parameters between WT and CXCR2 KO mice after saline infusion (Fig. 5A–C). Taken together, the above data suggest that CXCL1 is necessary for promoting the restoration of CXCR2^+^ macrophages in Ang II-induced retinopathy.

### 2.5 Blockage of CXCR2 rescues Ang II-induced retinopathy

**Fig. 6:**
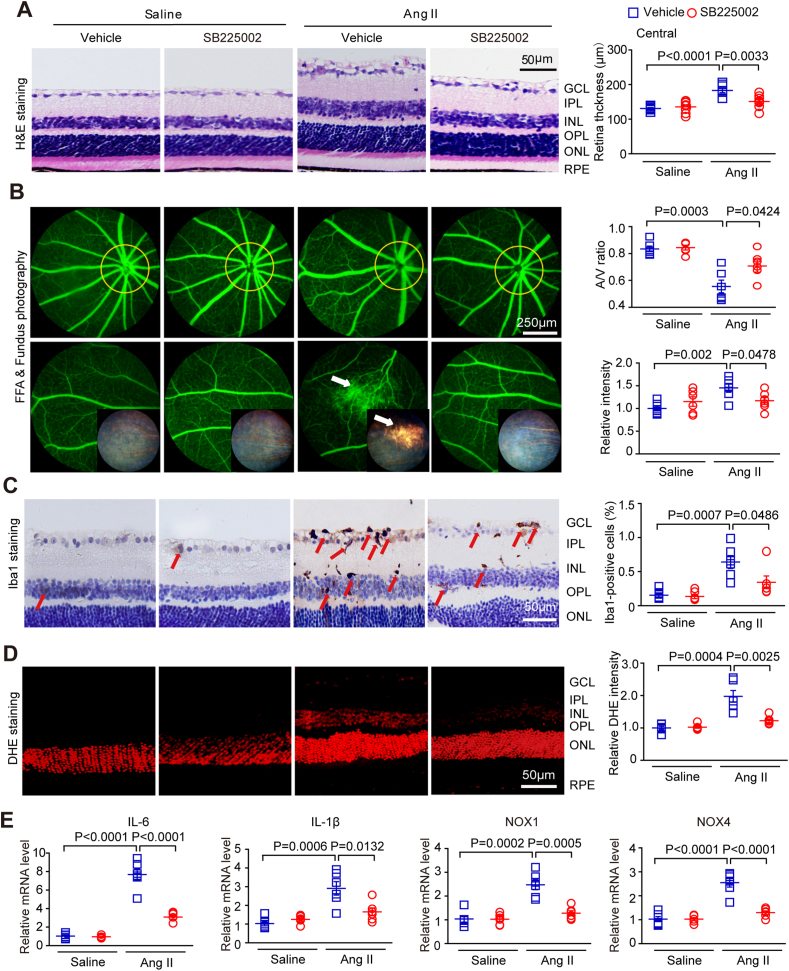
**The CXCR2 inhibitor SB225002 alleviates Ang II-induced changes in retinal thickening, inflammation, ROS, and vascular dysfunction.** (A) WT mice were treated with SB225002 or vehicle (DMSO) and then with angiotensin II infusion for three weeks. Central retinal sections stained with haematoxylin and eosin (left). Measurement of retinal section thickness (right, n = 8). (B) A representative retinal angiogram and fundus image (left); the white arrow indicates a fluorescein leak and the corresponding fundus image area. The ratio of retinal arteriovenous and fluorescence intensity was quantified (right, n = 6). (C) Representative images of Iba1 IHC staining, red arrows indicate the Iba1-positive microglia/macrophages(scale bar: 50 μm, left); quantification of positive cells in each group (right, n = 6). (D) Representative images of superoxide generation (left) and quantification of the fluorescence intensity (red) for DHE (right). (E) Analyses of IL-1β, IL-6, NOX1 and NOX4 transcript levels in the retina (n = 6). (For interpretation of the references to colour in this figure legend, the reader is referred to the Web version of this article.)

To evaluate whether CXCR2 is a promising therapeutic target for HR, we administered SB225002 (a specific inhibitor of CXCR2) to WT mice for 3 weeks. Our results showed that administration of SB225002 did not exert cytotoxic effects in the eyes of saline-treated mice, as determined by histology, and the SBP was comparable after saline or Ang II infusion (Fig. S3). Moreover, consistent with the findings in CXCR2 KO mice (Fig. 3), the Ang II-induced increase in central retina thickness, arteriolar morphological changes, infiltration of Iba1^+^ macrophages, and superoxide production were significantly blocked in SB225002-treated mice unlike in vehicle-treated mice (Fig. 6A–D). In addition, Ang II-induced upregulation of IL-1β, IL-6, NOX1, and NOX4 mRNA expression in the retinas of vehicle-treated mice was attenuated by SB225002 treatment (Fig. 6E), indicating that CXCR2 might be a new therapeutic target for HR.

### 2.6 Circulating CXCR2^+^ proinflammatory cell levels are increased in human hypertensive retinopathy

**Fig. 7:**
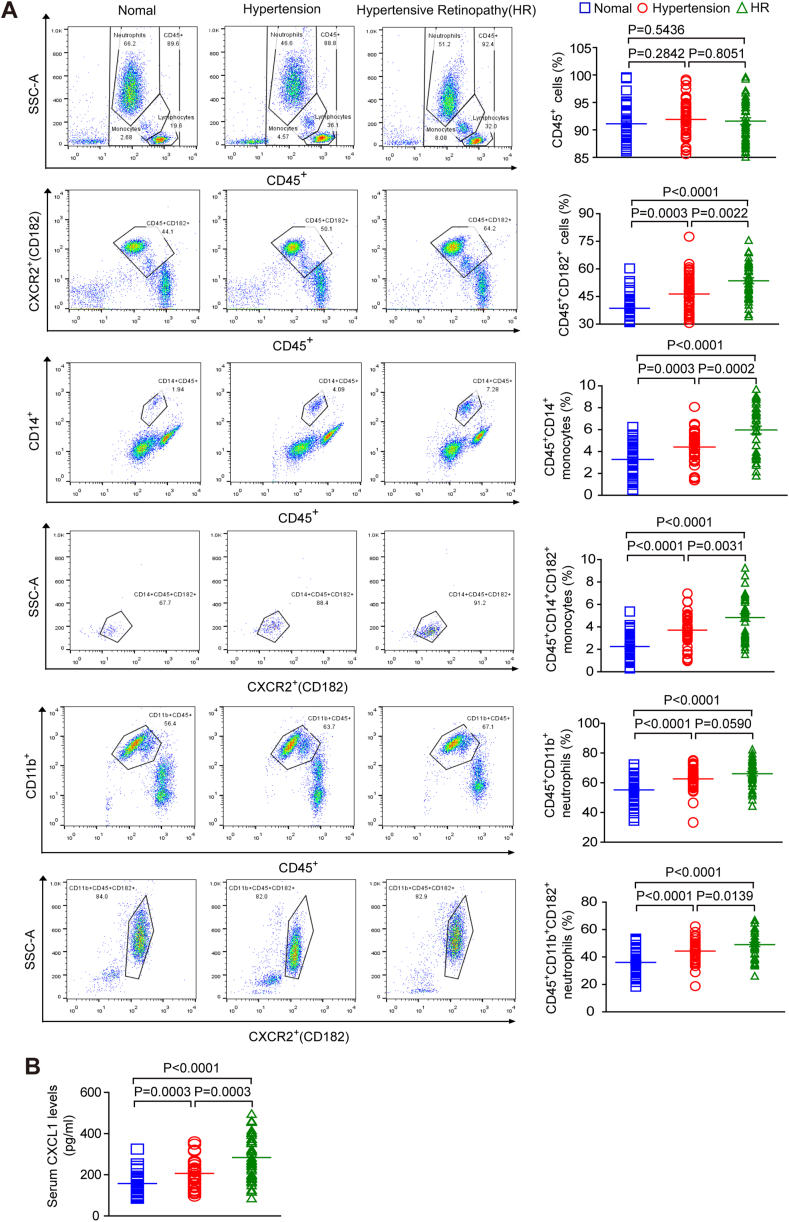
**The number of CXCL1 and CXCR2**^**+**^**immunocytes in the serum is elevated in patients with hypertensive retinopathy.** (A) Analysis of circulating immune cells based on flow cytometry, including CD45^+^ cells, CD45^+^CD182+ (CXCR2^+^) cells, CD45^+^CD14^+^ monocytes, CD45^+^CD14^+^CD182^+^ monocytes, CD45^+^CD11b^+^ neutrophils and CD45^+^CD11b^+^CD182^+^ neutrophils, in healthy controls (n = 40), hypertensive patients (HP) (n = 40) and hypertensive retinopathy patients (HR) (n = 40). (**B**) Analysis of serum CXCL1 levels in healthy controls (n = 40), HP patients (n = 40), and HR patients (n = 40) by ELISA.

To determine whether CXCL1 and CXCR2 are associated with HR in humans, we examined serum CXCL1 levels and the number of CXCR2^+^ immune cells as well as other related risk factors in healthy controls (n = 40) and patients with HR (n = 40) and hypertension (HP, n = 40). The baseline characteristics of all subjects are shown in Table 1. Patients with HP or HR had a higher SBP and higher total cholesterol, triglyceride, and LDL levels than healthy controls (Table 1). Flow cytometry revealed that the percentage of CD45^+^ immune cells, including CD182^+^ (CXCR2) cells, CD14^+^ monocytes, CD14^+^ CD182^+^ monocytes, CD11b^+^ neutrophils and CD11b^+^ CD182^+^ neutrophils, in the serum was significantly higher in the patients with HP or HR than in the normal controls (Fig. 7A). The ELISA results showed that serum CXCL1 levels were also higher in patients with HP or HR than in healthy controls (Fig. 7B). Furthermore, multivariable logistic regression models revealed a statistically significant relationship between the CXCL1 level and HP or HR (OR: 0.972, 0.987) and between the percentage of CD45^+^ cells, including CD182^+^ cells (OR: 0.843, 0.925), CD14^+^ CD182^+^ monocytes (OR: 0.117, 0.360), and CD11b^+^ CD182^+^ neutrophils (OR: 0.841, 0.952) and HP or HR after adjusting for sex, age, LDL levels and HDL levels (Table 2).

**Table 1:** Baseline Characteristics of healthy controls and patients with hypertension (HP) or hypertensive retinopathy (HR).

Parameters	Healthy controls (n = 40)	Patients with HP (n = 40)	Patients with HR (n = 40)
Ages, yrs	54 ± 10	58 ± 9	55 ± 10
Male	50%(20)	60%(24)	47.5%(19)
Systolic blood pressure (mm Hg)	126 ± 14	156 ± 14***	157 ± 11***
Diastolic blood pressure(mm Hg)	76 ± 8	91 ± 12***	93 ± 12***
Smoke	40%(16)	55%(22)	40%(16)
Alcohol drinking	42.5%(17)	50%(20)	32.5%(13)
**Examinations**			
Cholesterol (mmol/L)	3.987 ± 0.486	4.575 ± 1.120*	4.619 ± 1.157*
Triglycerides (mmol/L)	1.072 ± 0.287	1.596 ± 1.038*	2.033 ± 1.557*
HDL (mmol/L)	1.215 ± 0.274	1.152 ± 0.242	1.151 ± 0.286
LDL (mmol/L)	2.108 ± 0.447	2.520 ± 0.769*	2.432 ± 0.790*
Creatinemia (μmol/L)	76.901 ± 13.035	76.951 ± 26.518	69.975 ± 23.740
Fasting blood glucose (mmol/L)	5.221 ± 1.297	5.411 ± 1.703	5.312 ± 0.925
White blood cell count (10^9^/L)	6.422 ± 1.417	6.441 ± 2.135	6.521 ± 2.478

*P < 0.05, **P < 0.01, ***P < 0.001vs control. HDL, high density lipoprotein; LDL, low-density lipoprotein; ACEI, Angiotensin converting enzyme inhibitor; ARB, Angiotensin Receptor Blocker. The parameters are the mean ± SEM or n (%).

**Table 2:** Results of the multivariable logistic regression models of CXCL1 and CXCR2+ cells on HP and HR patients.

	Multivariate analysis		
	OR	95% CI	P-value
**Normotensive Controls**	1.000	reference	NA
**Hypertension Patients**			
CXCR2 CD45	0.843	0.759–0.936	0.001
CXCR2 CD45CD14	0.117	0.117–0.048	<0.001
CXCR2 CD45CD11b	0.841	0.749–0.945	0.004
CXCL1	0.972	0.956–0.988	0.001
**Hypertensive Retinopathy**			
CXCR2 CD45	0.925	0.858–0.996	0.040
CXCR2 CD45CD14	0.360	0.195–0.662	0.001
CXCR2 CD45CD11b	0.952	0.878–1.032	0.231
CXCL1	0.987	0.977–0.998	0.016

Data were expressed as odds ratio (OR) and 95% confidence interval (CI) adjusted by age, sex, HDL and LDL and calculated with logistic regression models.

## 3 Discussion

**Fig. 8:**
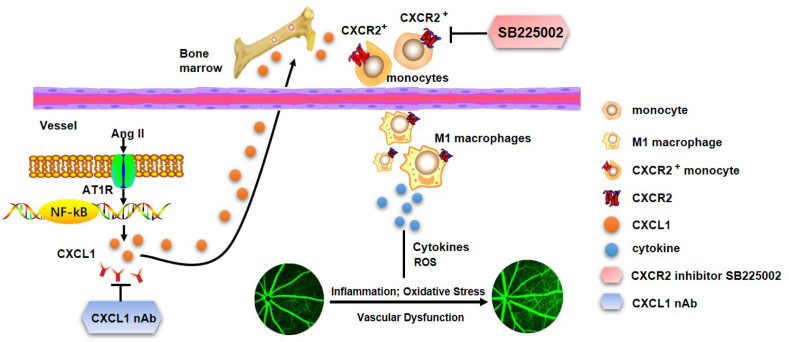
A working model of the mechanism by which CXCL1 recruits CXCR2^+^ macrophages into the retina. These macrophages initiate and aggravate Ang II-induced changes in central retinal thickness, arterial remodelling, inflammatory cell infiltration, ROS production and vascular dysfunction. Conversely, suppressing CXCL1 with neutralizing antibodies (nAbs) or CXCR2 with SB225002 alleviates these negative consequences.

We first demonstrated that CXCL1 expression was enhanced in the retinas of Ang II-treated mice, which subsequently induced CXCR2+ infiltration. Additionally, increased levels of circulating CXCR2+ monocytes were observed in HR patients. Pharmacological blockade of CXCL1 and CXCR2 or deletion of CXCR2 markedly attenuated Ang II-induced retinal structural remodelling, macrophage infiltration, proinflammatory cytokine expression, and oxidative stress and alleviated retinal dysfunction, as shown in Fig. 8. Accordingly, all the results in our study support an important causative role for CXCL1–CXCR2 in the pathogenesis of HR. Selective targeting of this signalling pathway could serve as a potential treatment for HR.

Monocyte recruitment is a feature of retinopathies, including macular degeneration (AMD) [[15], [16], [17]], retinitis pigmentosa [16], retinal detachment [18], oxygen-induced retinopathy [19], and diabetic retinopathy [20]. However, the potential mechanisms by which hypertensive stress drives the migration and infiltration of proinflammatory cells into the retina in early stages remain unclear. Chemokines are a large class of chemotactic cytokines that mediate the migration of monocytes/macrophages by binding to G protein-coupled receptors, thus leading to the production of various factors and reactive oxygen species and causing endothelial dysfunction [13,21]. Moreover, previous studies have shown that CXCR2 is mainly expressed on monocytes, macrophages and neutrophils but is also expressed on endothelial cells (ECs), retinal microglial cells, astrocytes, and Muller cells [[22], [23], [24], [25]]. Consistently, our results confirmed that CXCL1, CXCL3 and CXCL5 as well as CXCR2 were highly expressed in retinas after Ang II stimulation (Fig. 1A–C). Accordingly, the numbers of CXCR2^+^ macrophages and neutrophils were also markedly increased in Ang II-treated retinas and in the sera of patients with HR compared with the sera of controls (Fig. 1, Fig. 7A). These findings suggest that the increase in CXCL1 and CXCR2 expression may be involved in Ang II-induced retinopathy.

Recently, researchers have found that chemokines and their receptors are involved in the development of retinopathy [13,26]. For example, CCL5 is a potential systemic biomarker, and administration of a CCR2/CCR5 inhibitor (TAK-779) significantly reduces retinal vascular leakage in DR [27,28]. A CCR3 inhibitor (AKST4290) combined with intravitreal anti-VEGF injections has been used to treat exudative age-related macular degeneration (AMD) [29]. Several studies suggest that CXC chemokines, such as CXCL1, and their receptor CXCR2 are strongly associated with the migration and recruitment of monocytes/macrophages and neutrophils in different inflammatory diseases, including hypertension, cardiac remodelling, and atrial fibrillation [3,9,10,30]. A recent study indicated that administration of the CXCR2 antagonist SB225002 attenuates lens injury-induced cell infiltration through Akt activation [13]. However, it is unclear whether CXCL1-CXCR2 signalling participates in Ang II-induced retinopathy. To answer this question, we used CXCR2 KO mice and WT mice as experimental subjects. The mice were treated with the inhibitor SB225002 or anti-CXCL1 and the results showed that blocking CXCL1 and CXCR2 activity alleviated Ang II-induced retinal arteriolar remodelling, oxidative stress, and retinal dysfunction. Most importantly, blocking CXCL1 or knocking out CXCR2 significantly reduced the number of immune cells, particularly macrophages and neutrophils, and the expression of proinflammatory cytokines (IL-1β, IL-6, MCP-1 and TNF-α) and NADPH oxidases (NOX1 and NOX4) (Fig. 4, Fig. 5, Figures. S1C and S2D), demonstrating a critical role for CXCL1-CXCR2 signalling in regulating Ang II-induced retinopathy partially via mediation of proinflammatory cell infiltration and oxidative stress. Together, these data indicate that CXCL1 and CXCR2 may be new targets for the treatment of HR.

Increasing amounts of evidence indicate that Ang II plays an important role in the initiation and progression of vascular endothelial dysfunction, inflammation and vascular remodelling [31]. Ang II–induced effects of hypertension on retinal vessels lead to vascular changes, including intimal hyperplasia, hyaline degeneration, endothelial cell barrier dysfunction and breakdown of the blood–retina barrier (BRB), which promote exudation of serum proteins and lipids into the retina, leading to fluid accumulation (oedema) in multiple layers of the retina [[32], [33], [34]]. The blood‒retinal barrier (BRB) mainly comprises retinal pigment epithelium and retinal endothelial cells and retinal pigment epithelial (RPE) cells. The BRB is critical for maintaining the integrity of retinas [35]. It is generally accepted that disruption of the BRB is the main cause of retinal oedema and vision loss [35]. There are many factors that influence BRB permeability and retinal oedema formation. Tight junction proteins, such as ZO-1, can maintain ionic and metabolic gradients through the interaction of endothelial cells with pericytes and glial cells, which are crucial for the normal function of the BRB [36]. Moreover, vascular endothelial growth factor (VEGF) is a key factor contributing to BRB dysfunction in several eye diseases. An increase in VEGF levels causes BRB decomposition in diabetic retinopathy patients [37]. Furthermore, VEGF treatment causes a marked increase in fluid accumulation and hydraulic conductivity, leading to retinal oedema [38]. Consistently, the present results confirmed that Ang II infusion for 3 weeks also upregulated VEGF-A levels but downregulated ZO-1 expression in the retinas of WT mice (Figs. S2B–C), leading to vascular leakage and BRB dysfunction. Importantly, the Ang II-induced response was remarkably reversed in the retinas of CXCR2 KO mice (Figs. S2B–C), suggesting that CXCR2 KO not only attenuates the proinflammatory response but also alleviates vascular leakage and BRB breakdown, which protects against Ang II-induced retinopathy in mice.

There are several limitations in this study. The exact mechanism by which Ang II upregulates the expression of CXCL1 and CXCR2 in retina needs to be elucidated. Our study focused on the effect of CXCR2 expressed on monocytes/macrophages on Ang II-induced retinopathy. The contribution of neuronal and endothelial CXCR2 to hypertensive retinopathy remains to be explored. The role of CXCL1-CXCR2 signalling in regulating vascular leakage, retinal oedema and thickness needs more study in the future.

In conclusion, we are the first to identify a critical role for the CXCL-1-CXCR2 axis in mediating macrophage recruitment into the retina, leading to the retinal inflammatory response and oxidative stress during Ang II-induced retinopathy. Our findings may provide important insights for future clinical treatment because the expression of CXCL1 and the number of CXCR2+ immune cells are significantly increased in HR patients. Therefore, selective targeting of CXCR2+ monocytes/neutrophils may be a promising HR treatment. Further research is needed to verify the role of CXCL1-CXCR2 signalling in HR development in myeloid-specific CXCR2 KO mice and other animal models.

## 4 Material and methods

### 4.1 Animals and treatment

WT C57BL6J mice and CXCR2 KO mice (B6129S2 [C]-Cxcr2tm1Mwm/J) were purchased from the Jackson Laboratory (Sacramento, CA). All the animals needed adaptive feeding for 1 week before surgery. The retinopathy model was induced by subcutaneous infusion of Ang II (3000 ng/kg/min), and normal saline was infused as a control. Ang II infusion was achieved using osmotic micropumps (Alzet models 1007D and 1004, Durect, Cupertino, CA) for 1–3 weeks as described previously [5,6]. A specific inhibitor of CXCR2 (SB225002, Selleck, Houston, TX) was dissolved in vehicle (castor oil). An anti-CXCL1 antibody (100 μg/mouse/day) or SB225002 (2 mg/kg/day) was injected intraperitoneally (IP) into male mice (8–10 weeks old) one day before infusion of angiotensin II and for three weeks following as described previously [3,9]. The same volume of control IgG or vehicle (castor oil) was IP injected in the control group at the same time in addition to an anti-CXCL1 antibody (100 μg/mouse/day), whereas mice were treated with IgG in the control group. All animals were raised in specific pathogen-free (SPF) surroundings and given free access to drinking water and a normal diet. The experimental procedures used in this study were all approved by Dalian Medical University and according to the National Institutes of Health guidelines for the care and use of experimental animals (No. 85-23; Bethesda, Maryland, USA).

### 4.2 Systolic blood pressure recordings

As described previously, the SBP of the mice in each group was monitored with the tail-cuff system (BP-98A, Softron, Japan) [6]. The basal SBP of each mouse was measured one day before surgery, and SBP was monitored on the 3rd, 7th, 14th and 21st days after Ang II or Saline infusion.

### 4.3 Histological and immunohistochemical staining

All animals were euthanized. The eyecups were fixed in eyeball fixation fluid for more than one day. After being deparaffinized and rehydrated, paraffin sections of retina sections (4 μm) were prepared for use. For thickness measurements of retinas in each group, four sections from each eye at least 60 mm apart were subjected to H&E staining. The central retinal thickness was measured as 500 μm from the optic nerve head (ONH). Immunohistochemical staining was performed with an antibody against ionized calcium-binding adaptor molecule 1 (IBA1; 1:500; Abcam, ab178846) and then with a corresponding secondary antibody. The sections were treated with DAB, and the nuclei were counterstained with haematoxylin [39].

### 4.4 Immunofluorescence assay

The eyes of the mice in each group were fixed in 4% PFA for 4 h and then cryopreserved in 30% (w/v) sucrose solution overnight. The next day, the eyes were embedded in tissue OCT-freeze medium (APPLYGEN). For immunofluorescence, each group of eye sections was incubated with primary antibodies, including anti-CXCR2 (PA5-102662, 1:200; Invitrogen), anti-ZO-1 (ab216880, 1:200; Abcam), and anti-VEGFA (ab52917, 1:200; Abcam), at 4 °C overnight. On another day, all the sections of each group were incubated with Alexa Fluor 594-conjugated secondary antibody (Abcam, ab150080 1:200) for 1 h at room temperature (RT) and then with DAPI (Beyotime Biotechnology, C1002, 1:200) for 5 min at RT. For DHE staining, the eyes were directly embedded in OCT medium after being quickly removed from the mice. Fresh frozen eye sections (8 μm thickness) were incubated in a light-protected humidified chamber at RT with DHE (1 mM in PBS) for 30 min as described previously [39]. Four sections from each eye at least 60 mm apart were randomly selected for intensity quantitation. The four nonoverlapping areas of each section were magnified 200 times and analysed with ImageJ software.

### 4.5 Quantitative real-time PCR

TRIzol reagent (Invitrogen, New York) was used to isolate total RNA from fresh frozen retinas obtained from each group according to the manufacturer's instructions. First-strand cDNA (2 μg) was composited by using a Superscript II Kit (TAKARA, Japan). All the primers were composited by Invitrogen and are enumerated in Table S1. GAPDH was used as the internal control, and the levels of each mRNA expression were normalized to the expression level of GAPDH. Relative expression levels were calculated according to the ΔΔCt method.

### 4.6 Western blot assay

Total protein was extracted from rapidly frozen retinas by using RIPA buffer (Beyotime) and 1% protease inhibitor (PMSF, Beyotime) (RIPA:PMSF ratio of 1:100). Twenty-five micrograms of protein lysate from each sample was electrophoretically separated with a 10% SDS–PAGE gel and then transferred to a polyvinylidene fluoride membrane. Anti-CXCR2 (Invitrogen, PA5-102662, 1:200) and anti-GAPDH (Cell Signalling Technology, CST, D16H11, 1:1000) were used to incubate the membranes at 4 °C overnight and then with secondary antibodies. We developed all the blots with the ECL-Plus chemiluminescence system and used ImageJ software for densitometry analysis. GAPDH was used as an internal control.

### 4.7 Fluorescence angiography

Pupil dilation was achieved with 2.5% hydroxy methylcellulose ophthalmic-analgesic solution ophthalmic gel (Gonak, Akorn, Lake Forest, IL, USA) and tropicamide eye drops. Then, fluorescein sodium was administered by caudal injection, which was accomplished using an OPTO-RIS retinal imaging device (Optoprobe Science, Burnaby, British Columbia, Canada) that continuously imaged the retinal blood vessels for 5 min. Branching anatomy and pulsatile activity were used to identify the arteries. Each mouse was assigned a standard anatomical parameter, i.e., the diameter of the two optic discs, to calculate the arteriovenous ratio as described previously [40].

### 4.8 Electroretinography (ERG)

After being acclimatized to darkness for more than 24 h, the mice were anaesthetized, their corneas were desensitized with Novartis cornea desensitizer (Novartis Eye Hospital, Basel, Switzerland), and the pupils were dilated with a drop of 1% tropicamide. Animals were kept at 37.0 °C. The right cornea was connected to a gold wire electrode. The right forelimb was ground with a needle electrode. The left eye (unstimulated) was covered with a dark patch, and ERGs were recorded [6].

### 4.9 Flow cytometry

The mice were euthanized and perfused, and the retinas were removed and placed in RPMI-1640 (Sigma Aldrich) supplemented with 5% foetal bovine serum (FBS). The retinas were treated with 0.5 mg/mL collagenase D (Roche, Indianapolis, IN, USA) and 250 μg/mL DNase (Sigma Aldrich). The tissue was disrupted with a pipette in a microcentrifuge tube at 37 °C and then digested with 1 mL RPMI containing 5% FBS for 30 min. The digested tissue fragments were passed through a 70 μm cell filter with a 3-mL syringe plunger to obtain single-cell suspensions. The cells were washed twice with PBS-2% FBS supplemented with 1 mM EDTA (fluorescence-activated cell sorting buffer) and then incubated with a CD16/32 antibody at room temperature for 1 h. After antigen blocking, the suspended cells were incubated with CD45-BV510, CD11b-PerCP-Cy5.5, F4/80-BV421, Gr-1-APC-H7 and CXCR2-PE or negative control (BD Biosciences) at 4 °C for 30 min in the dark [41]. The suspended cells were then washed with PBS, resuspended, and analysed with a Fortessa flow cytometer and Cell Quest Software (BD Biosciences).

## 5 Patients and sample processing

An institutional ethics committee approved the protocol for the Second Affiliated Hospital of Dalian Medical University and confirmed that it complied with the Declaration of Helsinki standards. The study included 40 people with normal blood pressure, 40 with hypertension (HP) and 40 with hypertensive retinopathy (HR). All participants signed a written informed consent form. As described previously, the Keith–Wagner–Baker (KWB) classification was used for the diagnosis of HR patients [42]. We collected blood samples from participants through preoviduct venipuncture. K2EDTA tubes were used to inhibit blood clotting. The blood samples were placed in erythrolysis buffer (BD) and incubated with CD45-Percp-cy5.5, CD14-APC, CD11b-BV510 and CXCR2 (CD182)-FITC (BD) as described previously [10]. Based on the gating parameters, events were collected and analysed by flow cytometry (BD).

### 5.1 ELISA

Serum CXCL1 protein levels in patients were measured by ELISA (DGR00B, R&D Systems) according to the manufacturer's instructions. For analysis of mouse retinal CXCL1 levels, each retina was homogenized in 0.1% Triton X-100 in PBS supplemented with a cocktail of protease inhibitors (Invitrogen, Carlsbad, CA). The samples were cleared by centrifugation and then assessed for protein concentration with a BCA protein assay. CXCL levels were detected with an ELISA kit (GROα/CXCL1, Elabscience, E-EL-M0018c) according to the manufacturer's instructions. After the reaction was terminated with sulfuric acid, the absorbance of each well was read at a wavelength of 450 nm on a plate reader.

### 5.2 Statistical analyses

The statistical analysis was performed with SPSS software 19.0 (SPSS, Chicago, IL, USA). Each quantitative result is presented as the mean ± SEM. ANOVA and independent t tests were used to compare the data. The multivariable logit model was used to evaluate the relationship between HP/HR and the number of circulating CXCR2^+^CD45^+^ cells, CXCR2^+^CD45^+^CD14^+^ monocytes, CXCR2^+^CD45^+^CD11b neutrophils and CXCL1 concentrations. A *P* value of< 0.05 was considered to indicate a statistically significant difference.

## Author contributions

S.W. and J.B. performed research; J.B., S.W., Y.-L.Z., Q.-Y.L., X.H., W.-K. Q., P.-F. Z and Y–S. G analysed data; H.-H.L. Q.Z., and S.W. conceived and designed research; H.-H.L. wrote the paper; and all authors approved the manuscript.

## Declaration of competing interest

The authors declare no competing interests.

## Data Availability

Data will be made available on request.
