# Supplementary material for: CXCL1-CXCR2 signalling mediates hypertensive retinopathy by inducing macrophage infiltration
Source: Redox Biol. 2022 Aug 13;56:102438. doi: 10.1016/j.redox.2022.102438 (PMC9418605; doi:10.1016/j.redox.2022.102438)
Supplement: Multimedia component 1 [file mmc1.docx]

**Supplementary data**

**Table S1 Primers used for quantitative real-time PCR**

| Gene | Forward Primer (5’- 3’) | Reverse Primer (5’- 3’) |
| --- | --- | --- |
| CXCR2 | ACTACTGCAGGATTAAGTTTACCTC | TCTCTGAGTGGCATGGGACA |
| CXCL1 | ACCCAAACCGAAGTCATAGCC | TTGTCAGAAGCCAGCGTTCA |
| CXCL2 | CAGGCTACAGGGGCTGTTGT | ACATCAGGTACGATCCAGGC |
| CXCL3 | CCCAGACAGAAGTCATAGCCA | ACACATCCAGACACCGTTGG |
| CXCL5 | TCCTCAGTCATAGCCGCAAC | GCTTTCTTTTTGTCACTGCCC |
| IL-1β | CTTCCCCAGGGCATGTTAAG | ACCCTGAGCGACCTGTCTTG |
| IL-6 | GCTACCAAACTGGATATAATCAGGA | CCAGGTAGCTATGGTACTCCAGAA |
| TNF-α | ATGGCCTCCCTCTCATCAGT | CTTGGTGGTTTGCTACGACG |
| MCP-1 | ATGGCCTCCCTCTCATCAGT | ACCTTAGGGCAGATGCAGTTTTA |
| NOX1 | CCCATCCAGTCTCCAAACATGAC | ACCAAAGCTACAGTGGCAATCAC |
| NOX4 | CTTGGTGAATGCCCTCAACT | TTCTGGGATCCTCATTCTGG |
| GAPDH | GGTTGTCTCCTGCGACTTCA | GGTGGTCCAGGGTTTCTTACTC |


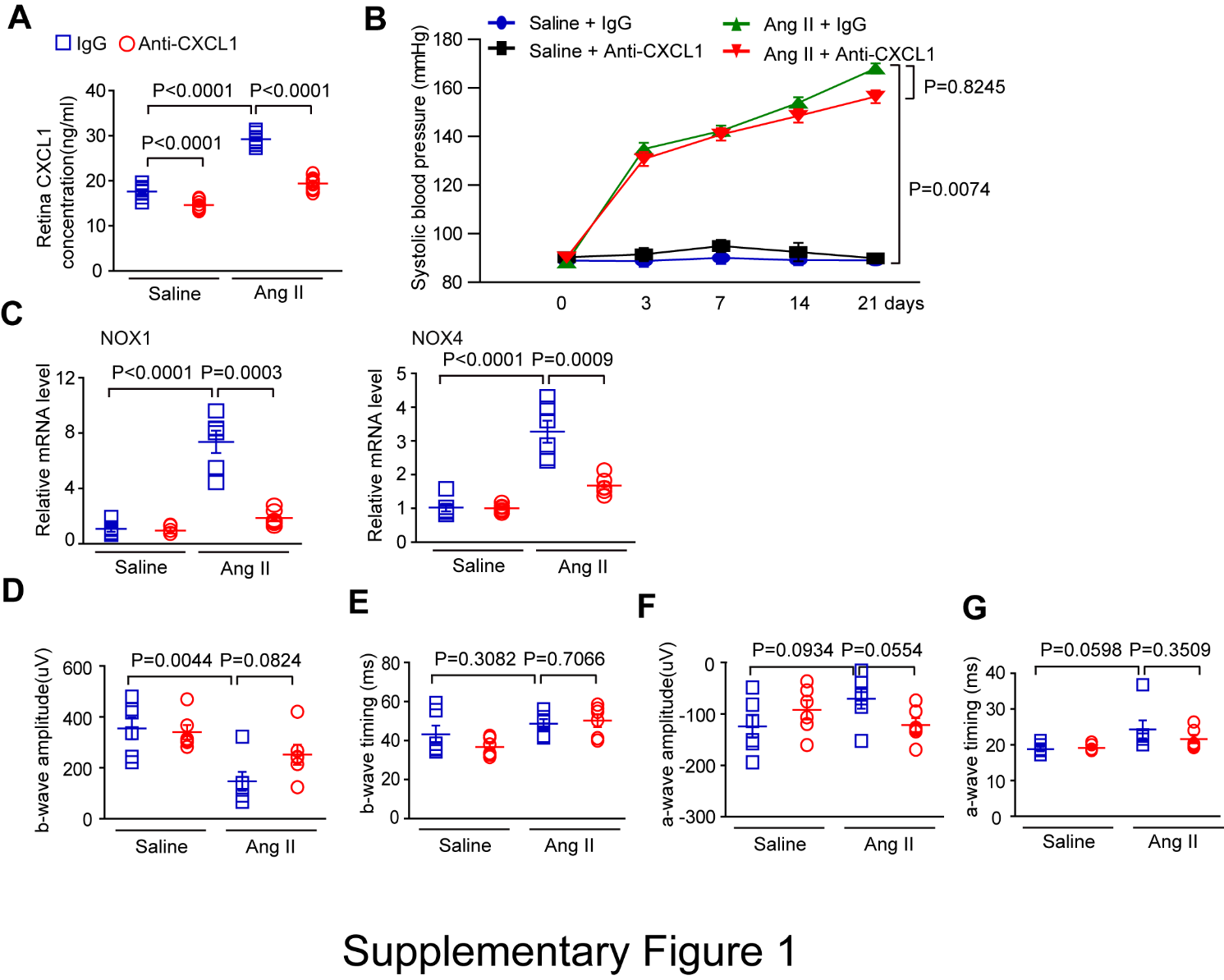


**Figure S1. The changes of SBP and the mRNA levels of CXCL1, NOX1 and NOX4 as well as the amplitudes of a-wave and b-wave of ERG in the retinas of mice after treatment with CXCL1 neutralizing antibody**

(A) Wild-type (WT) mice treated with IgG or anti-CXCL1 and then infused with saline or Ang II for 21 days. (B) ELISA assay of CXCL1 in the retinas (n=6 per group). (B) Measurement of systolic blood pressure (SBP) (n=8-10 mice per group). (C) qPCR analysis of the mRNA levels of NOX1 and NOX4 in the retinas (n=6). (D) Quantification of b-wave amplitude (n = 6 per group). (E) Quantification of b-wave timing (n = 6 per group). (F) Quantification of a-wave amplitude (n = 6 per group). (G) Quantification of a-wave timing (n = 6 per group). Results are the mean ± SEM.


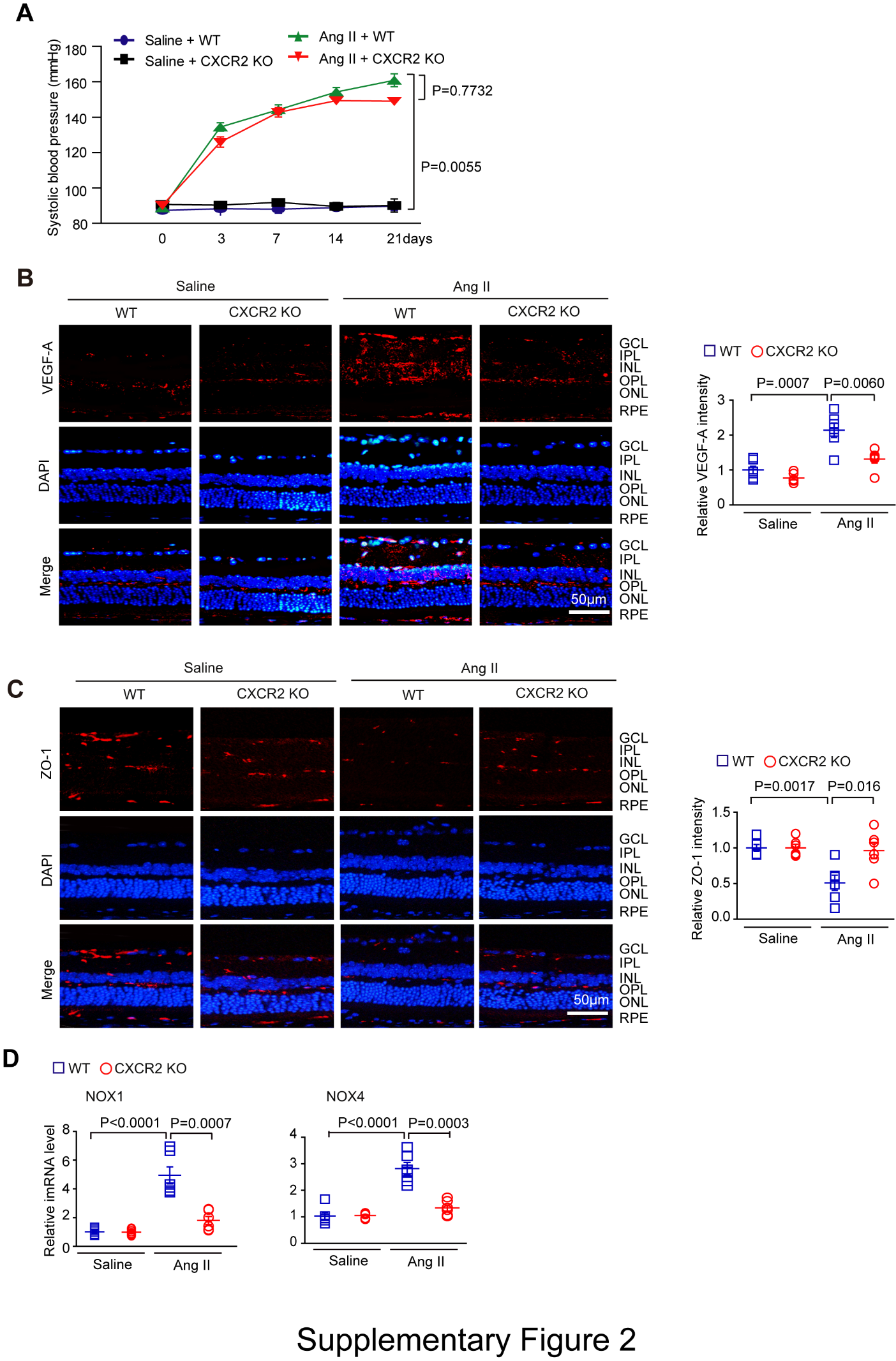


**Figure S2. Analysis of SBP, the expression of VEGF-A, ZO-1, NOX1 and NOX4 in the CXCR2 KO and WT mice**

(A) Wild-type (WT) mice and CXCR2 knockout (KO) mice were infused with saline or Ang II for 21 days. Measurement of systolic blood pressure (SBP) (n=8-10 mice per group). (B) Immunostaining of retinal sections with anti-VEGF-A antibody, and quantification of red fluorescence intensity (n = 6 per group). (C) Immunostaining of retinal sections with anti-ZO-1 antibody, and quantification of red fluorescence intensity (n = 6 per group). Nuclei were counterstained with DAPI (blue). Scale bars: 50 mm. (D) qPCR analysis of the mRNA levels of NOX1 and NOX4 in the retinas (n=6). Data are the mean ± SEM.


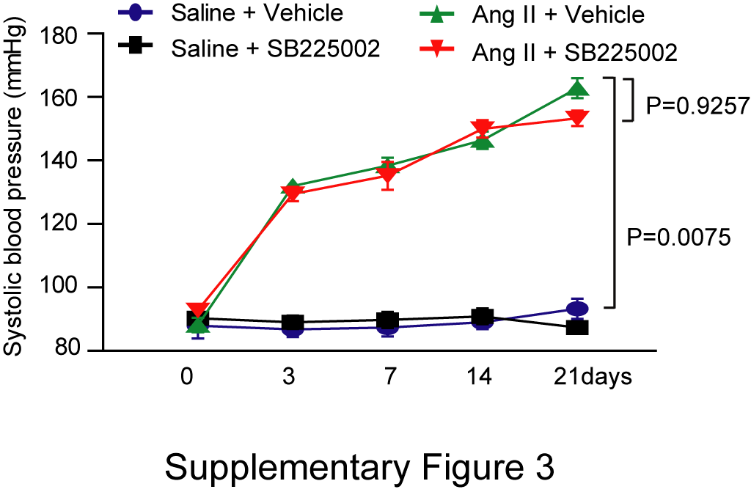


**Figure S3. Systolic blood pressure in mice with vehicle (DMSO) or CXCR2 inhibitor treated**

WT mice were treated with vehicle (DMSO) or SB225002, and then infused with saline or Ang II for 21 days. Measurement of systolic blood pressure (SBP) (n=8-10 mice per group). Data are the mean ± SEM.
